# MAOS and Medicinal Chemistry: Some Important Examples from the Last Years

**DOI:** 10.3390/molecules16119274

**Published:** 2011-11-07

**Authors:** Nailton M. Nascimento-Júnior, Arthur E. Kümmerle, Eliezer J. Barreiro, Carlos A. M. Fraga

**Affiliations:** 1 Laboratório de Avaliação e Síntese de substâncias Bioativas (LASSBio), Faculdade de Farmácia, Universidade Federal do Rio de Janeiro, PO Box 68023, Rio de Janeiro 21941-902, RJ, Brazil; 2 Programa de Pós-Graduação em Química, Instituto de Química, Universidade Federal do Rio de Janeiro, Rio de Janeiro 21949-900, RJ, Brazil; 3 Departamento de Química, Instituto de Ciências Exatas, Universidade Federal Rural do Rio de Janeiro, Seropédica 23890-000, RJ, Brazil; 4 Programa de Pós-Graduação em Farmacologia e Química Medicinal, Instituto de Ciências Biomédicas, Universidade Federal do Rio de Janeiro, Rio de Janeiro 21941-902, RJ, Brazil

**Keywords:** microwave irradiation, medicinal chemistry, bioactive compounds, drug discovery

## Abstract

This review aims to highlight microwave-assisted organic synthesis as applied to medicinal chemistry in the last years, showing some reactions performed under microwave irradiation for the synthesis of distinct structurally molecules of biological interest, divided into the following groups: antineoplastics, anti-inflammatory, antimicrobial agents, antivirals, agents for the treatment of neglected diseases and central nervous system-acting prototypes.

## 1. Introduction

The first reports describing the use of microwave irradiation in organic synthesis were published in 1986 by Gedye *et al.* and Giguere *et al.* [1,2]. After commercial microwave equipment became available (in the mid-1990s) the application of microwaves in organic chemistry has increased significantly, specially because of its positive effects, reducing reaction time and increasing yields. Consequently, problems related with reproducibility, safety and controlled conditions as temperature, stirring and pressure were solved, contributing to the development of the area of microwave-assisted organic synthesis (MAOS) [3]. In addition, new conditions, strategies and technologies were incorporated to MAOS, for example: solvent-free synthesis, which is an example of an eco-friendly approach [4], aqueous media reactions [5], multicomponent reactions [6], combinatorial chemistry [7], continuous flow reactions, microreactor reactions [8] and large scale reactions [9], showing that the problem related with the small amount of products obtained for pharmaceutical industry application is, maybe, about to be solved.

In the context of medicinal chemistry, a large variety of biologically interesting scaffolds obtained using MAOS is described in literature: indoles [10], quinoxalines, pyrido[2,3-*b*]pyrazines, thieno[3,4-*b*]pyrazines [11], quinazolines [12], [1,2,4]triazolo[4,3-*a*]pyridines [13], porphyrines [14], 1,2,4-triazoles [15], 1,2,4-oxadiazoles [16], *N*-acylhydrazones [17], the pyrazolo[4,3-*d*]pyrimidinone heteroaromatic subunit, present in the pharmaceutical drug sildenafil [18], *etc*. 

**Figure 1:**
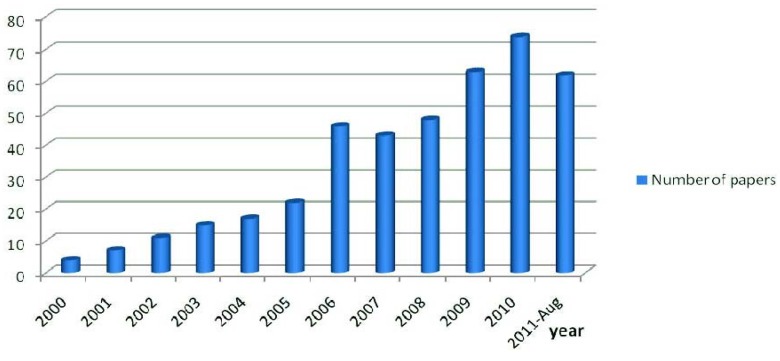
The number of papers published since the 2000th year (until August, 2011) in medicinal chemistry journals using the keyword “*microwave*” (www.scopus.com).

The growth in the number of papers published in the medicinal chemistry area using microwave irradiation as a tool can be observed in the *SCOPUS* database [19] (Figure 1). We used the keyword “**microwave**” (in article title, abstract or keywords) and the main medicinal chemistry journals: “*Journal of Medicinal Chemistry*”, “*European Journal of Medicinal Chemistry*”, “*Bioorganic and Medicinal Chemistry*”, “*Bioorganic and Medicinal Chemistry Letters*”, “*Molecules*”, “*ChemMedChem*”, “*Current Medicinal Chemistry*”, “*Journal of Pharmaceutical Sciences*” and “*Mini-Reviews in Medicinal Chemistry*”. These results show the progressive increase in interest in the application of MAOS in drug discovery, as can be observed in the medicinal chemistry literature [20,21,22].

To illustrate the application of MAOS in medicinal chemistry, we have selected various examples of reactions and scaffolds of biological interest that can be obtained using microwave irradiation. For this purpose, these reactions were distributed according to therapeutic class into six areas: antineoplastics, anti-inflammatory, antimicrobial agents, antiviral, neglected diseases and central nervous system.

## 2. Antineoplastics

The pathophysiology of cancer is one in which a group of cells shows uncontrolled growth, tissue invasion, and sometimes metastasis. Neoplastic cells can be divided into benign, where the events of invasion and metastasis do not occur, and malignant. Cancer can affect individuals of all ages, but is most prevalent in older people, being responsible for the death of 7.9 million people worldwide in 2007 [23].

Several research groups have sought to use inhibitors of kinases as a way to combat cancer. Among these, one can cite the EGFR tyrosine kinase (endothelial growth factor receptor), VEGFR (vascular endothelial growth factor receptor) and FGFR (vascular endothelial fibroblast growth factor receptor) and Bcr-Abl (target action imatinib), the PI3K (phosphatidylinositol 3’quinase), the cyclin-dependent kinases (CDK) and serine-threonine kinases such as MAPK (mitogen-activated protein kinase) [24].

Within the universe of serine-threonine kinases, Lindsley *et al.* decided to explore the synthesis and structure activity relationship of new selective allosteric inhibitors of Akt, a protein kinase B (PKB) recently described [25], using microwave irradiation for the construction of a chemical library of quinoxalines and pyrazinones from 1,2-diketone intermediates [26].

**Figure 2:**
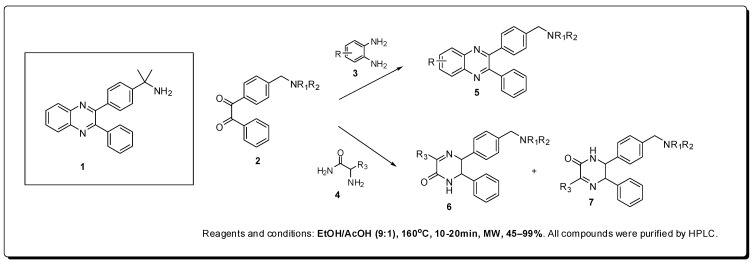
Synthesis of allosteric Akt kinase inhibitors with antineoplastic activity.

The authors used 2,3-diphenylquinoxaline **1**, a hit obtained by high throughput screening (HTS), and selective for isoforms 1 and 2, which are over expressed in several types of tumors, without action on PKA and PKC, as a prototype for the construction of its compounds (Figure 2).

The use of microwave irradiation through a scientific microwave reactor, allowed the rapid preparation of a library containing approximately 250 chemical compounds through reactions of various 1,2-diketones **2** with 1,2-diaminobenzenes **3** or substituted α-aminocarboxamides **4**, using a solvent mixture of EtOH/AcOH (9:1) at 160 °C for 10–20 min in yields ranging from 45 to 99%, after purification on preparative HPLC column.

**Figure 3:**
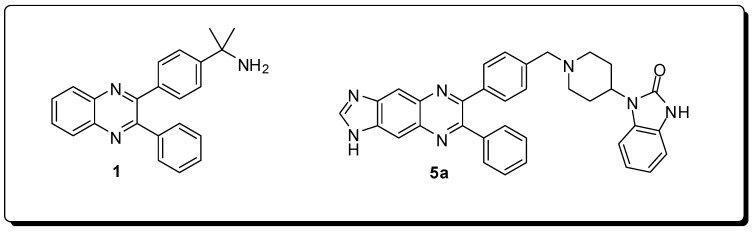
The diphenylquinoxaline **1** (*hit* obtained by HTS) and compound **5a**, compounds able to inhibit the *in vivo* phosphorylation of Akt.

The pharmacological evaluation of this chemolibrary demonstrated that several compounds were active on tumor cell lines, inhibiting allosterically the isoforms 1 and 2 of Akt, selectively, with potency in the nanomolar range. Moreover, compound **5a** (Figure 3) stood out, being able to inhibit the *in vivo* phosphorylation of Akt.

The PI3K-PKB-mTOR intracellular kinase signaling cascade is a major component in controlling cell proliferation and survival [27]. The misregulation of this pathway is primarily associated with the development of tumors in humans, in this case, leads to a superexpression of PKB, making it a target for therapeutic interventions of proven efficacy in animal models [28].

**Figure 4:**
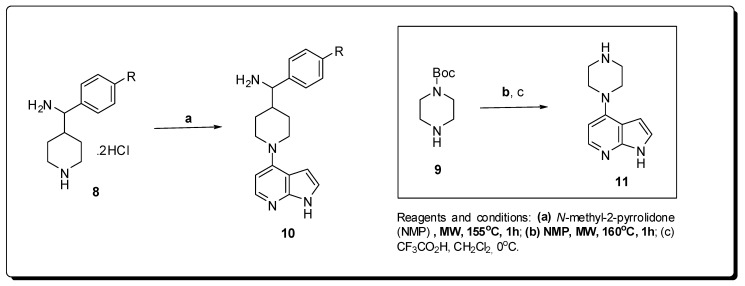
Microwave synthesis of pyrrolo[2,3-*d*]pyridine inhibitors of PKB.

Drug design based on crystallographic data structure, using chimeras of PKA and PKA-PKB, led to the discovery of new pyrrolo[2,3-*d*]pyridine compounds as selective inhibitors of PKBβ, with potency in the nanomolar range and PKB/PKA selectivity values around 30 [29]. The final step used in the construction of these compounds passed through a nucleophilic aromatic substitution reaction (S_N_Ar) of secondary amines **8** and **9** with functionalized pyrrolo[2,3-*b*]pyridine derivatives (Figure 4).

The reactivity in this step is very low under conventional heating, requiring drastic heating and long reaction times, in consequence, this reaction does not occur when less reactive amines are used. Through the use of a scientific microwave reactor, Caldwell *et al*. developed a new S_N_Ar methodology for halogenated purines which was then used in the synthesis of these PKBβ inhibitors [30].

Several recent studies have shown a correlation between cell cycle regulation and cancer. Thus, cell cycle inhibitors are being considered a tool for the management of cancer [31]. Notably, many groups have sought effective molecules that act on targets involved in phases G_0_/G_1_, such as cyclin D1, p53 and CdkIs for the control of inadequate cell proliferation [32].

**Figure 5:**
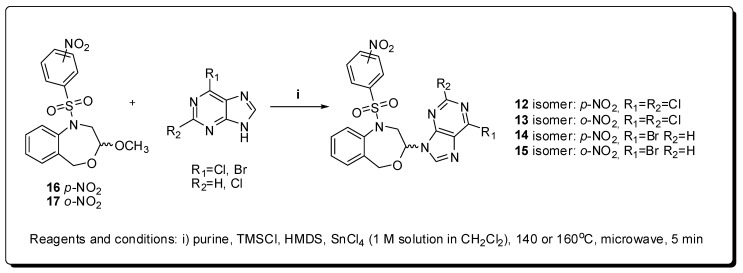
Preparation of the *O,N*-acetals anticancer derivatives **12**–**15**.

In this context, a complete SAR study was performed for a group of (*R,S*)-6-substituted-7- or 9-(1,2,3,5-tetrahydro-4,1-benzoxazepine-3-yl)-7*H* or 9*H*-purines for their anticancer activity [33]. The designed compounds **12**–**15** (Figure 5) were obtained by the microwave-assisted Vorbrüggen one-pot condensation of the cyclic acetals **16** and **17** [34] and the commercially available purine bases 6-chloro-, 6-bromo- and 2,6-dichloropurines, using chlorotrimethylsilane (TMSCl), 1,1,1,3,3,3-hexamethyldisilazane (HMDS) and tin(IV) chloride as the Lewis acid in anhydrous acetonitrile in a scientific microwave reactor. Their antiproliferative activities on MCF-7 and MDA-MB-231 cancerous cell lines led to the discovery of an active enantiomeric mixture (*RS*)-2,6-dichloro-9-[1-(*p*-nitrobenzenesulfonyl)-1,2,3,5-tetrahydro-4,1-benzoxazepin-3-yl]-9*H*-purine (**12**) that presented an IC_50_ of 0.166 μM against the human cancerous cell line MDA-MB-231.

## 3. Anti-Inflammatory

The inflammatory process is a response to an aggression caused by an external agent and can be divided into acute and chronic inflammation, according to the time required to resolve it [35]. In this context, there are several targets for the treatment of inflammation and inhibition of pro-inflammatory cytokines, like the tumor necrosis factor alpha (TNF-α) and interleukins (IL-6 and IL-1β), that can be used for the treatment of rheumatoid arthritis, inflammatory intestinal diseases, medulla transplant-related rejection problems [36] and inflammatory diseases on the respiratory level [37].

**Figure 6:**
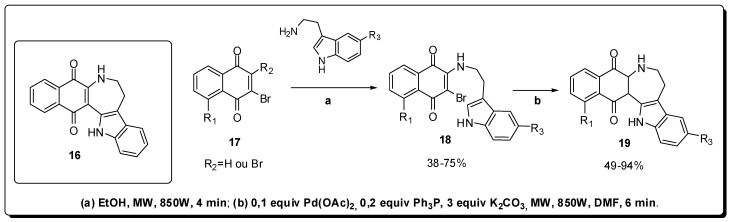
Synthesis of azepinonaphthoquinones with anti-inflammatory activity.

Based on the anti-inflammatory effects of quinones obtained from natural products [38,39], Phutdhawong and co-workers have planned and synthesized a series of 1,6,7,8-tetrahydronaphtho[2,3-*d*]azepino[4,5-*b*]indole-9,14-diones **16** (Figure 6) with inhibitory effects related with the pro-inflammatory cytokine production [40].

The synthetic route exploited to obtain the azepinonaphthoquinones **19** was based on the nucleophilic substitution reaction in bromonaphthoquinones **17** [41], followed by Pd-catalyzed intramolecular cyclization of **18**, using a domestic microwave oven. The products were obtained after extremely short reaction times, in good yields and without formation of reduction side products in the cyclization step (Figure 6). These results were important for the construction of this new series of heterocyclic derivatives that was able to reduce the production of pro-inflammatory cytokines *in vitro* assays.

**Figure 7:**
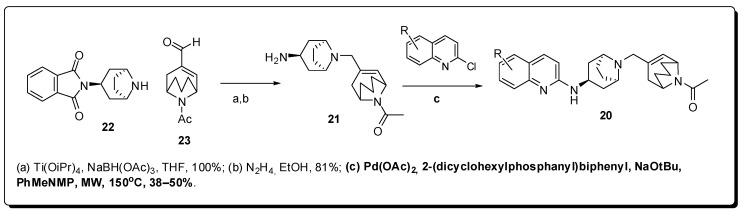
Anti-inflammatory 2-aminoquinolines obtained using MAOS.

Another anti-inflammatory approach consists in the antagonism of CXCR3 chemokine receptors, that are expressed mainly in T-lymphocytes (CD4^+^ and CD8^+^), B-lymphocytes, natural killer cells and astrocytes [42,43]. The antagonism of CXCR3 receptors are important to treat rheumatoid arthritis [44,45], multiple sclerosis, transplant rejection [46] and chronic obstructive pulmonary disease (COPD) [47]. For this propose, Knight and co-workers have described recently a series of 2-aminoquinolines **20** (Figure 7) with potent antagonist activity in CXCR3 and adequate physico-chemical properties, that have resulted in good bioavailability and *in vivo* activity [48].

The aminotropanic derivative **21** used in the Knight work was obtained through the reductive amination between the amine **22** and the aldehyde **23**, followed by hydrazinolysis. To obtain the derivatives **20**, it was used a microwave-assisted amination reaction between **21** and the corresponding chloroquinolines, using 2-(dicyclohexylphosphanyl)biphenyl as ligand, resulting in the formation of the desired product **20** in moderate yields (38–50%) [49].

## 4. Antimicrobial Agents

Since the antibacterial sulfonamide drugs were introduced into clinical practice in 1936 (the beginning of antimicrobial therapy) great advances have been achieved in the chemotherapy of infectious diseases. After World War II, important antimicrobial agents were found, e.g., penicillin, and nowadays this therapeutical class is composed by an expressive diversity of drugs. The recognition of replication mechanisms in bacteria, fungi or virus have helped in the rational development of compounds able to interfere with some key steps of vital cycle of these microorganisms [50]. In addition, the strategy of using genomic information for the identification and characterization of new biotargets has improved the discovery process of novel antimicrobial prototypes targeting resistant strains of different types of microorganisms [51].

The *N*-acylhydrazones and *N*-arylhydrazones classes are described in the literature as presenting antimicrobial, anticonvulsant, analgesic, anti-inflammatory, antitumoral, antiplatelet (antiaggregant), antineoplastic and other activities [52,53]. In this context, Ajani and co-workers have described MAOS of a series of 2-quinoxalinone-3-hydrazone derivatives expected to exhibit antimicrobial profile [54].

**Figure 8:**
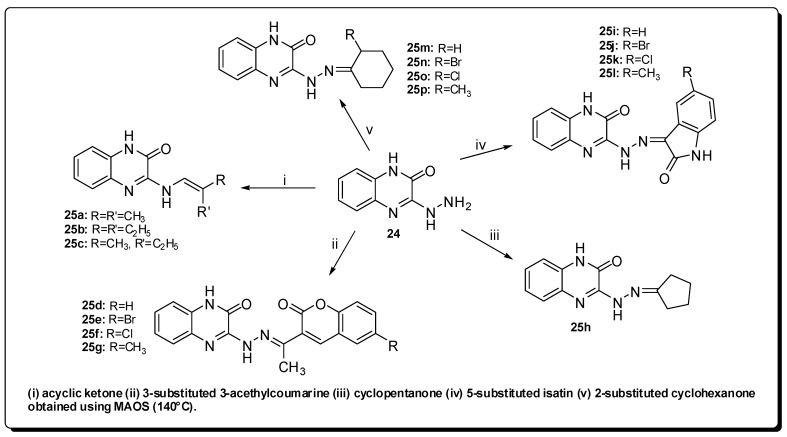
Synthesis of derivatives **25a**–**p**.

The condensation of the key-intermediate 3-hydrazinoquinoxalin-2(*1H*)-one (**24**) with the corresponding ketone derivatives, by using a scientific microwave reactor, has resulted in the formation of hydrazone derivatives **25a**–**p** in very short times (1–3 minutes) and excellent yields (55–99%) (Figure 8) [54].

It was observed that *E. coli* and *K. pneumoniae* have developed resistance against streptomycin, while all the synthesized derivatives **25a**–**p** were active against these two microorganisms. The evidenced inhibition zone is from 10 to 32 mm and the derivative **25f** stood out, showing the largest inhibition zone. Moreover, compound **25a** presented more pronounced antifungal activity against *C. albicans* [54].

The pyrazolines are well described in literature as having analgesic [55], anti-inflammatory [56], antibacterial [57], antifungal [57] and antitumoral [58] properties. As another examples of the use of MAOS applied to medicinal chemistry, Manna and co-workers described the synthesis of 1,3,5-tri-substituted indophenazyl-pyrazoline derivatives **30a**–**n** and their antimicrobial activity against multi-resistant bacteria [59].

**Figure 9:**
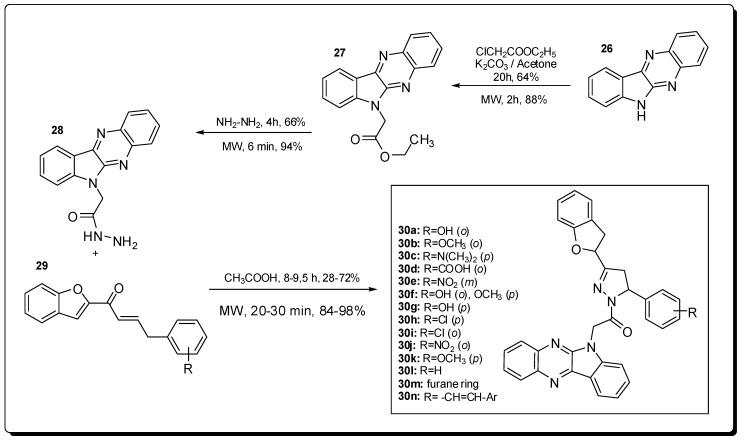
Synthesis of pyrazoline derivatives **30a**–**n**.

The preparation of derivatives **30a**–**n** was conducted in a domestic microwave oven following the synthetic route shown in Figure 9. For this example, reaction time and yields are much better by using microwave irradiation in the place of conventional heating [59].

Excepting compounds **30d** and **30m**, all 1,3,5-trisubstruted indophenazyl-pyrazoline derivatives **30a**–**n** showed good activity against *S. pyogenes*. The minimum inhibitory concentrations (MICs) of derivatives **30a**–**n** are adequate when compared with standard fluoroquinolone drugs. Additionally, the derivatives **30a**, **30e**, **30j**, **30k** and **30l** have shown the best antimicrobial activities against *E. coli*, *P. aeruginosa*, *S. typhi* and *S. aureus* [59].

## 5. Antivirals

**Figure 10:**
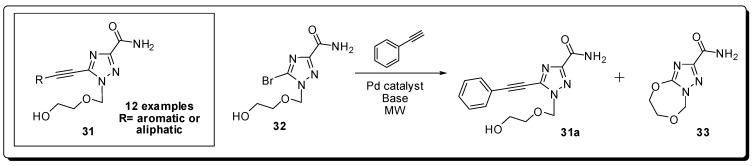
Synthesis of a series of compounds designed against the hepatitis C virus.

Among the diseases caused by viruses, hepatitis C is one of the most serious public health problems, affecting about 3% of the World’s population, which represents about 170 million infected people [60]. The current treatment against hepatitis C virus (HCV) consists in the use of pegylated interferon-α in combination with ribavirin [61]. However, this therapy is only effective in about 50% of the patients and is associated with serious side effects [61]. Therefore, it is necessary to find out more efficacious and better tolerated antiviral lead compounds. For this purpose, Zhu and colleagues have designed novel acyclic 1,2,4-triazole nucleosides **31** with various ethynyl moieties appended on the triazole nucleobase [62] (Figure 10).

The desired molecules were synthesized efficiently, in yields ranging from 75 to 99% starting from bromotriazole acyclonucleoside **32** using an efficient one-step Sonogashira reaction in aqueous solution and under microwave irradiation using a scientific microwave reactor. To avoid the formation of the intramolecular cyclization byproduct **33** under basic conditions, optimized conditions [Pd(PPh_3_)_4_/CuI and Li_2_CO_3_, in dioxane/H_2_O (3:1, v/v) as solvents for 25 min at 100 °C in a sealed vessel] were developed. One of the compounds (R = *para*-fluorphenyl) inhibited HCV subgenomic replication with a 50% effective concentration (EC_50_) of 22 μg/mL and did not inhibit proliferation of the host cell at a concentration of 50 μg/mL [63].

Another target identified as a binding partner for the hepatitis C Virus envelope glycoprotein E2 (HCV-E2) is the *large extracellular loop* (LEL) of the human CD81 cell surface protein, a member of the tetraspanin family [64]. Since inhibition of this interaction prevents HCV from infecting hepatocytes, the HCV principal target cells [64], Holzer *et al.* aimed to prepare compounds which restrain the CD81-LEL-HCV-E2 interaction by binding to the LEL [65]. His group started the work by using a biological screening of natural products, current drugs and their in-house substance library (approximately 350 compounds, including several structurally different antihistamines) using a medium throughput assay [66]. Terfenadine (**34**) was found to be a moderate inhibitor of the CD81-LEL-HCV-E2 interaction (27% at 50 μM).

**Figure 11:**
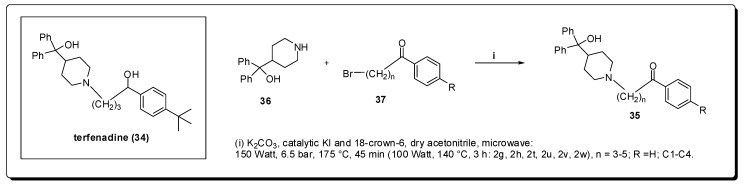
Synthesis of terfenadine analogues.

Precursors **37** were coupled to azacyclonol to furnish the planned terfenadine analogues **35** in good yields and short time (5–45 minutes) using MAOS [67] (Figure 11). The obtained ketoaryl-piperazine derivatives **35** presented an optimization regarding to the CD81-LEL-HCV-E2 interactions that resulted in an inhibitory profile of 69% at 50 μM.

**Figure 12:**
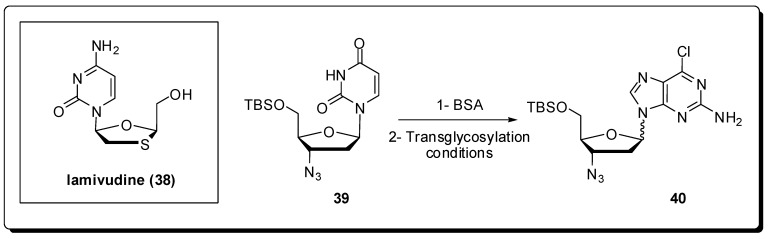
Lamivudine chemical structure and the reaction used for the optimization of the transglycosylation reaction with 3-azido nucleosides.

Regarding the anti-HIV therapy, the research for novel nucleoside analogues with high potency, low toxicity, and favorable resistance profiles [68] continues to be the cornerstone of this branch of anti-viral drug development [69]. In 1964, the first synthesis of an l-nucleoside was reported [70]; however, only natural D-nucleosides were assumed, during decades, to exhibit biological activity due to the stereospecificity of enzymes in living systems [71,72]. As a consequence, little attention was paid to l-nucleoside analogues until the 1990s with the discovery of lamivudine (**38**) that exhibited potent antiviral activity against HIV-1 and HBV [73,74,75] (Figure 12).

**Figure 13:**
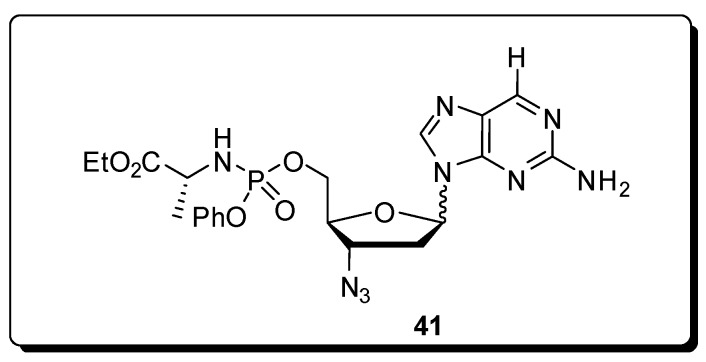
Chemical structure of the L-AZA prodrug **41**.

Zang *et al.* have described a rapid, simple, and general microwave assisted transglycosylation reaction for the synthesis of several azidopurine nucleosides presenting both D- and L-configuration [76]. They examined the effect of different Lewis acids as SnCl_4_, TMSI, TMSOTf, and Ti(O*i*-Pr)_4_ with potential to affect the transglycosylation of **39** to **40**. SnCl_4_ was found to be incompatible with **39** and led to formation of complex mixtures, whereas the use of Ti(O*i*-Pr)4 resulted in no reaction. When TMSI was utilized, derivative **40** was obtained in low yields as a 1.2:1 mixture isomers α and β. TMSOTf provided an 1:1 anomeric mixture of **40**, in yields higher than any other Lewis acid tested. Based on the reported microwave-assisted Vorbrüggen glycosylation reaction which was optimized to a 5 min ribosylation reaction at 130 °C [77], they began their studies with variation of the reaction temperature using a scientific microwave reactor. The stability of the azido group to TMSOTf at elevated temperature was evaluated and the best yields with minor degradation of **40** were obtained at 105 °C and 120 °C with 10 minutes of heating and 2.7 equiv. of TMSOTf. The synthesized compounds were evaluated for antiviral activity against both HIV and the L-AZA prodrug **41**, demonstrating significant anti-HIV activity with an EC_50_ value of 1.4 μM (Figure 13).

## 6. Neglected Diseases

One of the most neglected diseases in the World is the Human African Trypanosomiasis (HAT or sleeping sickness), which is caused by two subspecies of the parasite *T. brucei*, namely *Trypanosoma brucei rhodesiense* (causing East African sleeping sickness) or *Trypanosoma brucei gambiense* (causing West African sleeping sickness). Annually, approximately 50,000 people are reported with this disease and it is estimated that more than 300,000 are infected, but have not been diagnosed or treated [78,79,80,81,82].

**Figure 14:**
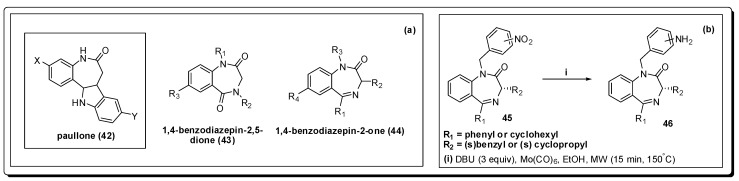
(**a**) Structures of paullone and benzodiazepines; (**b**) Reduction methodology used to obtain the corresponding amino compounds.

There is an urgent need for more effective treatments for HAT, as the current therapy has many shortcomings, *viz*. high toxicity, prohibitive costs, undesirable routes of administration as well as poor efficacy. The parallel synthesis of a series of 1,4-benzodiazepin-2,5-diones **43** (Figure 14), structurally related to the paullone nucleus **42**, was recently reported [83] as presenting antileishmanial and antitrypanosomal activities [84]. Due to the structural similarity of this series with 1,4-benzodiazepine-2-ones (BZDs), the synthesis and biological evaluation of a chemolibrary of the latter and their evaluation as antitrypanosomal agents showing moderate to good trypanocidal activity was described.

Microwave synthesis was used in order to create the *N*-benzyl series of analogues that presented moderated activity against trypanosomes [85]. The nitro compounds **45** was exploited as precursor of the corresponding anilines **46**, in yields ranging from 74 to 99%, by using molybdenum hexacarbonyl and DBU at 150 °C in a sealed tube for 15 min under microwave irradiation [86,87].

Malaria is a major cause of death, illness and poverty for approximately half of the world’s population [88]. It is a parasite-inflicted disease, which is spread throughout the tropical regions of the world by female mosquitoes of the genus Anopheles. Four species of protozoal parasites of the Plasmodium genus cause malaria in humans, *i.e.*, *Plasmodium falciparum*, *Plasmodium malariae*, *Plasmodium ovale* and *Plasmodium vivax*. Despite of considerable scientific advances and the development of new drugs, malaria is reported to cause over one million fatal cases annually, the majority being children under the age of five [88]. The constant emergence of parasitic strains resistant to the currently available antimalarial agents implies an acute need for new therapeutic agents with novel routes of action [89].

**Figure 15:**
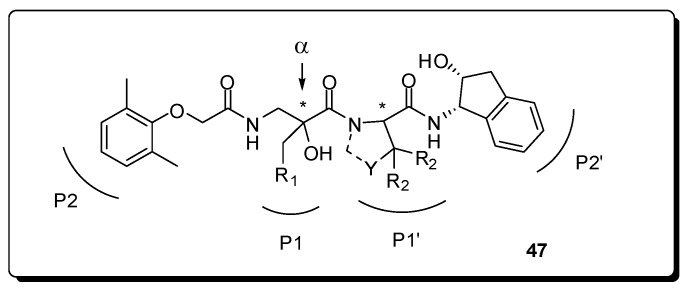
Generic structure of the designed α-substituted norstatine inhibitors with variable length of the tether to the P1-phenyl group, variations in the P10-group and two tunable stereocenters (*).

Twelve α-substituted norstatines **47** were designed (Figure 15), synthesized and evaluated for their inhibitory potencies against plasmepsin II and the plasmepsin IV orthologues (PM4) present in the digestive vacuoles of all four Plasmodium species causing malaria in man. The best compounds provided *K_i_* values in the nanomolar range for all PM4, with a best value of 110 *n*M in PM4 from Plasmodium ovale [90].

**Figure 16:**
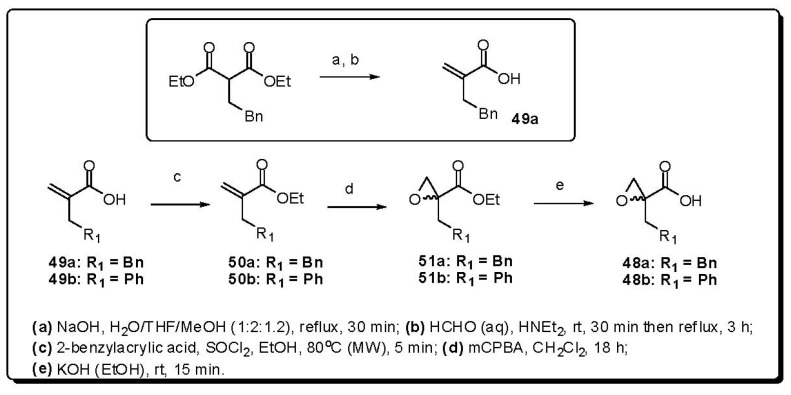
Synthesis of the P1 moieties.

The tertiary alcohol building blocks reported for the construction of α-benzylnorstatines and α-phenylnorstatines are prepared from the respective epoxy acids **48a** or **48b**. The novel, four-step synthetic route to these key intermediates is outlined in Figure 16. The acrylic acid **49a** was prepared by a Knoevenagel reaction, by applying a similar procedure to that previously reported for the synthesis of **49b** [91]. Attempts to perform the direct epoxidation of **49a**,**b** were not successful. Thus, a fast and convenient one-pot microwave irradiation (MW) protocol for esterification was developed, in order to generate the corresponding ethyl ester **50a**,**b**. The esters were then readily oxidized by 3-chloroperbenzoic acid (mCPBA) to provide the epoxides **51a**,**b**. The building blocks, **48a**,**b**, were obtained by hydrolysis of the ester.

**Figure 17:**
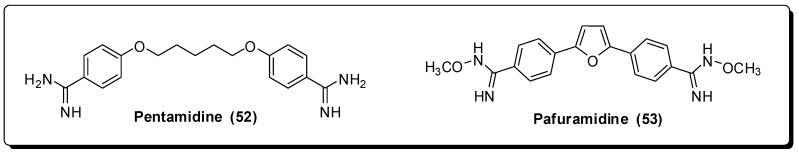
Compounds with antileishmanial activity.

Leishmaniasis is caused by protozoan parasites belonging to the genus *Leishmania* and is one of the most widespread parasitic diseases been endemic in 89 countries and its visceral form, caused by *Leishmania donovani*, leads to 5,000,000 new cases and 50,000 deaths per year [92]. Commonly a well-known antiprotozoan aromatic diamidine, pentamidine (**52**, Figure 17) [93], is used against various infectious diseases such as leishmaniasis [94,95], HIV-related *Pneumocystis jirovecii* opportunistic pneumonia [96] and trypanosomiasis [94,97]. Despite of its wide antimicrobial spectrum, its use remains limited due to its high toxicity, in particular nephro-, cardio- and neurological toxicities [93,98].

Arylamidines are known to bind to the minor groove of AT DNA sequences along the phosphodiester backbone [99,100]. Two amidinium end groups appear to be necessary for this interaction [101] while the central part of the drug inserts into the minor groove. However, the amidine group presents poor oral bioavailability due to its protonation in the biophase needing parenteral administration. Pafuramidine (**53**, Figure 17) has shown interesting activities against *L. donovani* and presented good oral bioavailability owing to the replacement of amidines by methoxyamidoximes **52** [102].

**Figure 18:**
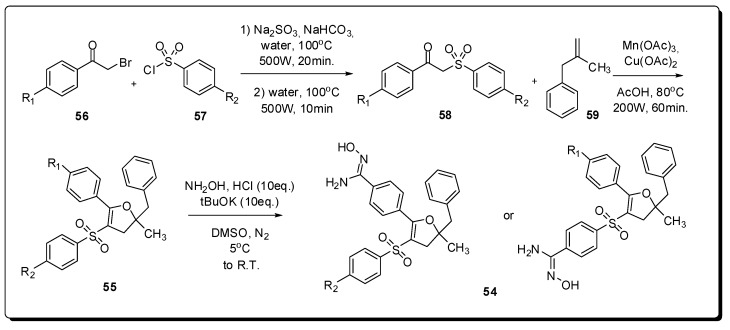
New series of monoamidoxime derivatives.

A new series of monoamidoxime derivatives **54** was designed by Paloque and colleagues and synthesized using manganese(III) acetate by means of microwave irradiation using a multimode microwave oven, specifically in the synthesis of the 2,3-dihydrofuran intermediates **55** from several β-ketosulfones **56** (Figure 18) [103]. Some amidoximes showed interesting *in vitro* activities toward *Leishmania donovani* promastigotes with IC_50_ values between 5.21 and 7.89 μM. Additionally, the cytotoxicity of the synthesized compounds was evaluated revealing the corresponding selectivity index (SI), where four amidoxime derivatives **54** have exhibited an SI more than 20 times better than pentamidine (**52**).

## 7. Central Nervous System

The central nervous system (CNS) drug market accounted for 15% (around U$ 118 billion) of the pharmaceutical market sales in 2008 [104]. In contrast with the neglected diseases, the major part of CNS diseases affects people of all countries and economical classes. In this context, there are many efforts to develop novel drug candidates, for example: with hypnotic and sedative effects [105], as alternative for the treatment of neuropathic pain [106] and other analgesic effects [107].

**Figure 19:**
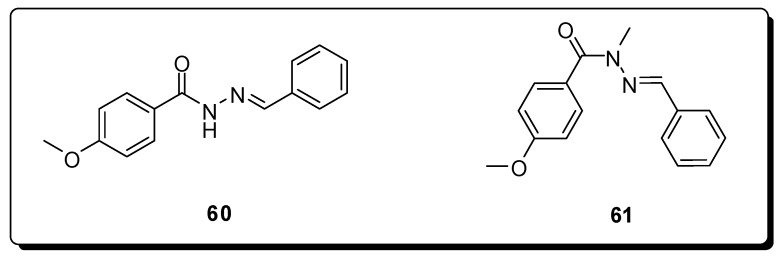
*N*-acylhydrazones with analgesic activity.

Among the compounds of *N*-acylhydrazones class developed in our research group, the major part of them presents both, analgesic and anti-inflammatory profiles [53]. Recently, we have discovered two compounds (**60** and **61**, Figure 19), that have inhibited the abdominal constrictions induced by AcOH in mice in 50% and ED_50_ of approximately 2 mg/Kg, for both compounds [107].

**Figure 20:**
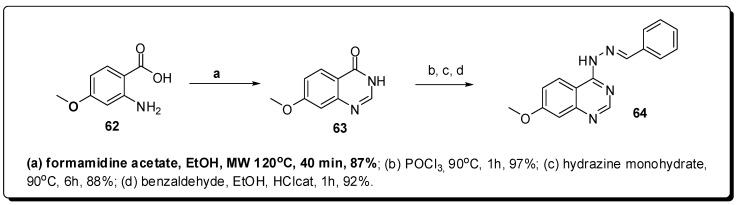
Use of MAOS to construct quinazoline scaffold.

Based on this results, were proposed conformational restrictions of **60** and **61**, as a strategy to elucidate the bioactive conformations, constructing heterocyclic rings mimetic to *N*-acylhydrazone framework. A scientific microwave reactor was used to construct three heterocyclic conformationally-restricted derivatives [107]. For the construction of quinazoline scaffold of **64** (Figure 20), MAOS allowed the synthesis of the quinazolinone intermediate **63** from the reaction between anthranilic acid **62** and formamidine acetate in good yield (87%). In this case, conventional heating resulted in the formation of **63** in lower yields.

**Figure 21:**
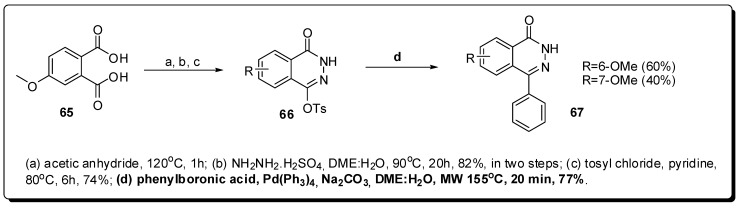
Use of MAOS to construct phthalazinone scaffold.

The second conformationally-restricted heterocyclic compound **67** was constructed through the Suzuki cross coupling between the phenyl boronic acid and the tosyl phthalazone **66**, resulting in the formation of **67** in only 20 minutes and good yield (77%) (Figure 21) [107].

**Figure 22:**
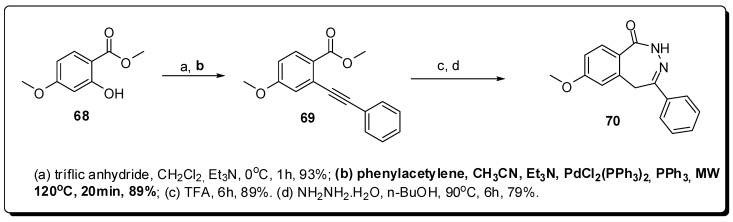
Use of MAOS to construct benzodiazepinone scaffold.

The third conformationally-restricted heterocyclic compound, a benzodiazepinone (**70**), were obtained through a Pd-catalyzed cross coupling, but in this case, the Sonogashira cross coupling was used to obtain the acetylenic derivative **69** (Figure 22). The use of MAOS was important for the reduction of the reaction time required the obtain **69**, *i.e**.***, 20 minutes at 120 °C (Figure 22), which was next cyclized to form **70** [107].

The antinociceptive effect of *N*-acylhydrazone derivatives **60** and **61** in AcOH-induced abdominal constrictions in mice induced by acetic acid was better than the effect observed for **64**, **67** and **70**. However the three conformationally-restricted derivatives presented analgesic potencies equivalent to paracetamol used as standard drug [107].

Another good example of MAOS applied to medicinal chemistry from our laboratory is related with the synthesis of four heterotricyclic neuroactive compounds **75a**–**d**. They were evaluated in hypnotic, locomotor and analgesic activity protocols [108,109]. In the pentobarbital-induced-sleep test [110], the nitro-derivative **75d** has shown an important hypnotic behavior with duration of approximately 160 minutes at 6 mg/Kg dose. On the locomotor activity test in mice [111], the compounds **75a** (6 mg/Kg, 93.7 ± 15.2 mov/min) and **75d** (4 mg/Kg, 86.7 ± 16.5 mov/min) stand out as being more active to promote a sedative effect, statistically equivalent to the reference drug midazolam [108,109,112] in a dose of 2 mg/Kg. The analgesic activity was evaluated in the hot plate test in mice [113] and the pyrazolo[3,4-*b*]pyrrolo[3,4-*d*]pyridine derivative **75d** presented the best analgesic activity (100% in the dose of 2 mg/Kg) after only 30 minutes.

**Figure 23:**
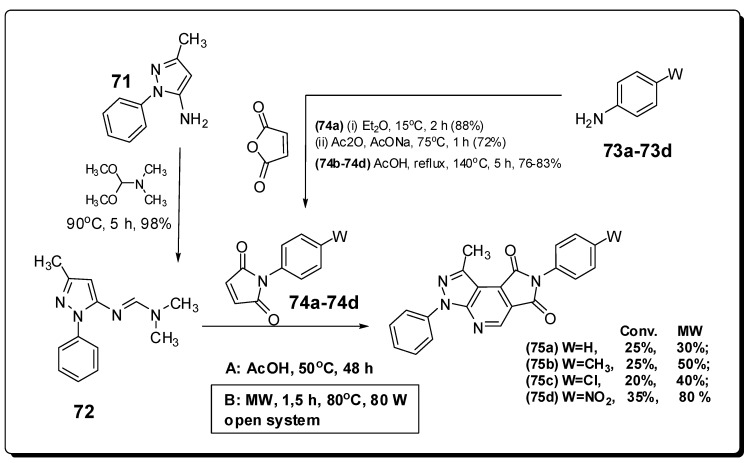
Synthesis of the intermediates **72** and **74a**–**d** followed by the hetero Diels-Alder step exploited to obtain the pyrazolo[3,4-*b*]pyrrolo[3,4-*d*]pyridine derivatives **75a**–**d**.

Before the construction of the four heterotricyclic compounds **75a**–**d**, the azadiene **72** was prepared in excellent yield (98%), through the reaction between the phenylpyrazolamine **71** and the *N*,*N*-dimethylformamide dimethylacetal. In addition, the phenylmaleimides **74a**–**d** were constructed by the condensation of *para*-substituted anilines **73a**–**d** with maleic anhydride in yields ranging from 72% to 83% [108,109,114]. The pyrazolo[3,4-*b*]pyrrolo[3,4-*d*]pyridine derivatives **75a**–**d** were constructed in a key cycloaddition step between the azadiene **72** and functionalized phenylmaleimides **74a**–**d**, in very low (20–35%) yields and very long reaction times (48 hours) when conventional heating was used (Figure 23) [108,109,114]. 

Based on the literature of MAOS applied to the synthesis of other heterocyclic compounds obtained by cycloaddition strategy [115,116,117], we have developed a new methodology to construct the pyrazolo[3,4-*b*]pyrrolo[3,4-*d*]pyridine derivatives **75a**–**d** in a scientific microwave reactor using a new free-solvent methodology. For the four heterotricyclic derivatives **75a**–**d** the reaction time was reduced from 48 hours to 1.5 hours and in the case of the heterocyclic compound **75d**, the yield was increased from 35 to 80% [114].

## 8. Conclusions

The extensive use of microwave irradiation for the synthesis of molecules of pharmacological interest has contributed to improve the access to different chemical scaffolds by applying new methodologies and techniques. For this reason, the initial limitations faced in MAOS seem to be disappearing and these facts encourage its use as the first option for the synthesis of new drug candidates, specially based on the benefits related to better yields and shorter reaction times. As could be exemplified in this review, there are research groups using MAOS to find out hits and lead-compounds related with different biological targets and diseases in the last years. In spite of the scale up limitations in MAOS, continuous flow technique and large reactors use have achieved good results in the challenge to obtain the amounts of substance required by the pharmaceutical industry for pre-clinical and clinical phases of the drug development process.
